# Pulsed field ablation of refractory alternating atrial fibrillation and atrial flutter using Impella CP in a patient with cardiogenic shock: a case report

**DOI:** 10.1093/ehjcr/ytaf216

**Published:** 2025-04-28

**Authors:** Isabel Rattka, Giulio Mastella, Eimo Martens, Karl-Ludwig Laugwitz, Manuel Rattka

**Affiliations:** School of Medicine and Health, Department of Clinical Medicine—Clinical Department for Cardiology, University Medical Centre, Technical University of Munich, Ismaninger Straße 22, D-81675 Munich, Germany; School of Medicine and Health, Department of Clinical Medicine—Clinical Department for Cardiology, University Medical Centre, Technical University of Munich, Ismaninger Straße 22, D-81675 Munich, Germany; School of Medicine and Health, Department of Clinical Medicine—Clinical Department for Cardiology, University Medical Centre, Technical University of Munich, Ismaninger Straße 22, D-81675 Munich, Germany; European Reference Network Guard Heart, European Union, Ismaninger Straße 22, D-81675 Munich, Germany; School of Medicine and Health, Department of Clinical Medicine—Clinical Department for Cardiology, University Medical Centre, Technical University of Munich, Ismaninger Straße 22, D-81675 Munich, Germany; German Centre of Cardiovascular Research (DZHK), partner site Munich, Ismaninger Straße 22, D-81675 Munich, Germany; School of Medicine and Health, Department of Clinical Medicine—Clinical Department for Cardiology, University Medical Centre, Technical University of Munich, Ismaninger Straße 22, D-81675 Munich, Germany

**Keywords:** Atrial fibrillation, Heart failure, Impella CP, Cardiogenic shock, Case report

## Abstract

**Background:**

Supraventricular tachycardia (SVT) can lead to cardiogenic shock, particularly in patients with severely impaired left ventricular function. Acute management typically includes electrical cardioversion combined with antiarrhythmic drugs to restore sinus rhythm. In cases of therapy-resistant SVT, atrioventricular nodal ablation may be considered, although this results in permanent pacemaker dependency. Definitive treatments, such as atrial fibrillation (AF) or atrial flutter (AFLUT) ablation, which could potentially avoid pacemaker implantation, are seldom pursued in such critical settings, despite advancements in therapeutic efficacy.

**Case summary:**

We present the case of a 79-year-old male with ischaemic cardiomyopathy and advanced heart failure, admitted with cardiogenic shock and regular narrow-complex tachycardia. Initial attempts at electrical cardioversion combined with pharmacological rhythm control provided only temporary success, followed by recurrent episodes of alternating AF and AFLUT. This prompted the decision to perform pulmonary vein isolation (PVI) and posterior wall isolation (PWI) using pulsed field ablation (PFA), supported by the Impella CP device for mechanical circulatory support. The procedure, guided by the CARTO 3 electroanatomic mapping system, successfully achieved PVI and PWI with restoration of sinus rhythm. Following the procedure, the patient’s haemodynamics stabilized, and sinus rhythm was maintained, along with significant improvement in left ventricular function.

**Discussion:**

This case underscores the feasibility of PFA-assisted PVI and PWI with Impella CP in the management of acute AF/AFLUT-induced cardiogenic shock. It highlights the role of mechanical circulatory support in enabling successful SVT ablation in haemodynamically unstable patients, offering an alternative to atrioventricular node ablation in critical situations.

**Learning points**

- In patients with cardiogenic shock due to therapy-resistant atrial fibrillation and atrial flutter, pulmonary vein isolation (PVI) and posterior wall isolation (PWI) by pulsed field ablation can safely be performed under Impella CP support.
- Pulsed field ablation for PVI/PWI does not interfere with Impella CP function.

## Introduction

Supraventricular tachycardia (SVT), including atrial fibrillation (AF) and atrial flutter (AFLUT), can lead to acute heart failure and cardiogenic shock, particularly in patients with severely impaired left ventricular function.^1,2^ In cases of haemodynamic instability, emergency electrical cardioversion is the preferred treatment. However, cardioversion may sometimes be ineffective, and SVT can recur, even with the concurrent use of antiarrhythmic drugs (AADs).^1,3^ Atrioventricular nodal (AVN) ablation is an effective treatment option in such situations.^4^ Nevertheless, AVN ablation is a definitive approach, requiring permanent pacemaker implantation afterwards. The standard therapy for haemodynamically stable patients—AF and/or AFLUT ablation—is rarely performed in those who are haemodynamically unstable, despite the availability of fast and effective treatment options today. We present the case of a patient in cardiogenic shock with AF/AFLUT, refractory to both pharmacological and electrical cardioversion, who was successfully treated with pulmonary vein isolation (PVI) and posterior wall isolation (PWI) using pulsed field ablation (PFA), supported by an Impella CP device.

## Summary figure

Timeline of the patient’s hospital stay. (*A*) Intraprocedural fluoroscopy, (*B*) surface electrocardiogram and intracardiac tracings, (*C*) post-procedural electroanatomic map.

**Figure:**
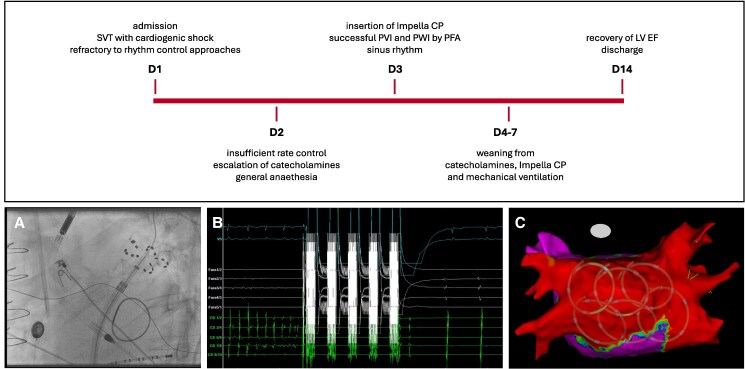


## Case presentation

We report the case of a 79-year-old Caucasian man with a history of advanced heart failure due to ischaemic cardiomyopathy, and mitral valve reconstruction.

In 2024, he was admitted to our emergency department by medical emergency services, presenting with shortness of breath, palpitations, and hypotension. On admission, he exhibited regular narrow-complex tachycardia at a rate of 125 beats per minute and a blood pressure of 73/42 mmHg. Left ventricular ejection fraction (LVEF) was 15%. Moreover, the patient had a serum lactate of 2.8 mmol/L and a serum creatinine of 1.6 mg/dL (reference level: 0.7–1.3 mg/dL).

Due to haemodynamic deterioration, direct current cardioversion was attempted, initially restoring sinus rhythm and stabilizing the patient. However, AF recurred within minutes, necessitating repeated electrical cardioversion. Because of alternating regular narrow-complex tachycardia and AF, continuous intravenous landiolol was administered for rate control. Additionally, intravenous amiodarone (initial dose of 150 mg followed by 1000 mg over 24 h per day) and catecholamines (noradrenaline 0.1 µg/kg/min) were initiated. On the second day, the patient’s haemodynamic status worsened, requiring increased catecholamine support. Multiple attempts at direct current cardioversion failed to achieve sustained sinus rhythm. Serum lactate rose to 4.3 mmol/L, the serum creatinine was 2.9 mg/dL, and the patient presented with anuria, indicating progressive cardiogenic shock.

Due to the patient’s haemodynamic instability and the need for repeated electrical cardioversion, general anaesthesia was induced. On the third day after admission, given the progression of heart failure and the increasing doses of nordadrenaline (0.7 µg/kg/min), we decided to proceed with an invasive ablation procedure using Impella CP for mechanical circulatory support (MCS), which had already been planned due to the deteriorating haemodynamics. A CT scan of the femoral arteries revealed significant kinking of the left femoral artery, prompting us to implant the Impella CP via the right femoral artery under ultrasound and fluoroscopic guidance. After successful implantation, the support mode was set to auto, generating an output of 3.2 L per minute.

**Figure 1:**
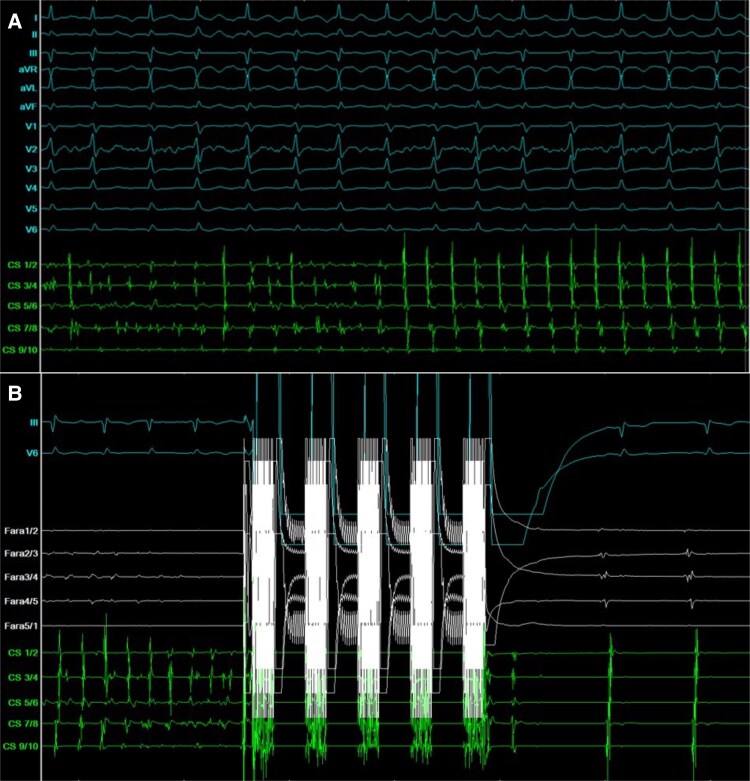
Surface electrocardiogram and intracardiac tracings. (*A*) Intracardiac tracings from the coronary sinus (CS) catheter show atrial fibrillation with irregular activation converting to a regular distal (CS 1/2) to proximal (CS 9/10) CS activation sequence. (*B*) Following energy delivery from the pentaspline pulsed field ablation catheter, atrial fibrillation converts to sinus rhythm.

For the ablation procedure, a decapolar catheter was first positioned in the coronary sinus. Intracardiac tracings revealed frequent variations in coronary sinus activation, indicating the presence of AF and left atrial re-entry tachycardia (*Figure 1A*). To minimize the duration of the procedure, we opted to perform PVI and PWI using PFA with a pentaspline catheter (Boston Scientific, MA, USA). Following a fluoroscopy-guided transseptal puncture and the introduction of the steerable FARADRIVE™ sheath (Boston Scientific, Menlo Park, CA, USA), an electroanatomic map of the left atrium was created using a high-density multi-electrode mapping catheter (OCTARAY™, Biosense Webster, Irvine, CA, USA) and the CARTO 3 mapping system (V7, Biosense Webster, Irvine, CA, USA).

**Figure 2:**
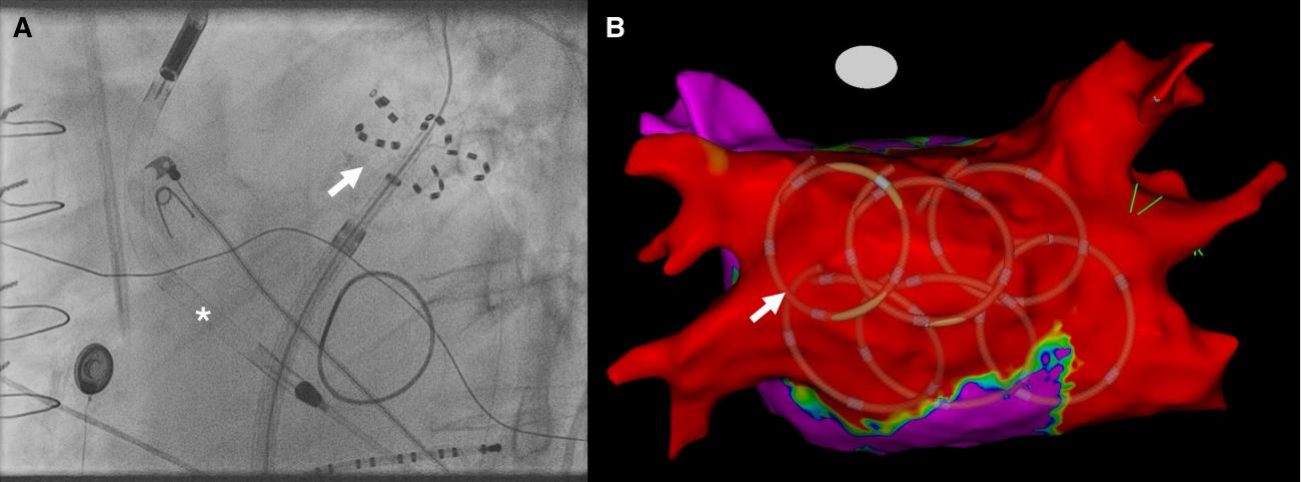
Intraprocedural fluoroscopy and post-procedural electroanatomic map. (*A*) Intraprocedural fluoroscopy shows the pentaspline pulsed field ablation catheter in flower configuration at the left superior pulmonary vein (white arrow). The Impella CP device (white asterisk) provides mechanical circulatory support. (*B*) Posterior view of the left atrium; the post-procedural electroanatomic map confirms successful pulmonary vein isolation and posterior wall isolation. The shadows (white arrow) mark the position of the ablation catheter during posterior wall isolation.

The 31 mm over-the-wire multipolar ablation catheter (FARAWAVE™, Boston Scientific, Menlo Park, CA, USA) was then introduced into the left atrium. Pulmonary vein isolation was performed with four applications in basket and four in flower configuration per pulmonary vein, as appropriate (*Figure 2A*). After PV isolation, two additional anchor lesions per vein were delivered in flower configuration with posterior torque on the steerable sheath. Posterior wall isolation was achieved by targeting the area between the left and right pulmonary veins, using overlapping applications in flower configuration without rotation. The catheter was displayed via impedance-only mapping, and lesion sets were planned by manually annotating the positions on the posterior wall. A total of six applications was sufficient to target the posterior wall, with a total of 46 PFA deliveries made.

During the procedure, AF converted to sinus rhythm (*Figure 1B*). Remapping confirmed successful PVI and PWI (*Figure 2B*). Towards the end of the procedure, Impella support was reduced. The total procedure time, including Impella CP insertion, was 121 min, with a left atrial dwell time of 48 min and an ablation time of 22 min from the first to the last energy delivery. Fluoroscopy time was 34 min with a radiation dose of 1680 cGy/cm^2^. No contrast dye was used.

Following the procedure, the patient was transferred to the intensive care unit. Over the following days, the Impella CP was removed, catecholamines were discontinued, and the patient was weaned off mechanical ventilation. Throughout the remainder of his hospitalization, the patient maintained sinus rhythm, and his LVEF improved to 38% upon hospital discharge. At the 6-month follow-up appointment, the patient was still in sinus rhythm. The transthoracic echocardiography showed a LVEF of 41%.

## Discussion

In patients with therapy-resistant atrial tachyarrhythmia-mediated cardiogenic shock, treating the underlying rhythm disorder is crucial to improving patient outcomes. When electrical cardioversion and/or the administration of AADs fail, atrioventricular (AV) node ablation is a valid treatment option. For instance, Hékimian *et al*. reported outcomes from seven patients with arrhythmia-induced cardiomyopathy who required venoarterial extracorporeal membrane oxygenation (VA-ECMO) and underwent AV-node ablation. Following AV-node ablation, LVEF improved in all patients, and ECMO was successfully weaned off.^5^

However, in addition to AV-node ablation, definitive treatment of SVT by ablation offers another option to restore haemodynamic stability when non-invasive therapies have been exhausted, and it can be safely performed with adjunctive mechanical support.^6^ Notably, studies have shown that in patients with AF and heart failure with reduced ejection fraction, PVI is associated with improved symptoms, a longer 6-min walk distance, and higher ejection fraction compared to patients who underwent AV-node ablation with subsequent biventricular pacing.^7^ Based on this evidence, we decided to proceed with SVT ablation with Impella CP support.

Suspecting AF and left atrial flutter, confirmed by intracardiac tracings, we opted for PVI and PWI using PFA with a pentaspline catheter to minimize procedure time. The ablation procedure was completed without complications, and AF converted to a normofrequent sinus rhythm during the intervention. In terms of our treatment strategy, we decided to perform PWI in addition to PVI because it appeared to be safe, fast, and feasible using the pentaspline PFA catheter, and because we believed that this lesion set would address more of the potential flutter mechanisms than PVI alone.^8^ However, it is possible that left atrial flutter might not have terminated, as an anterior line is often required to treat left atrial flutter in ablation-naïve individuals.^8^ In such a case, we would have switched to conventional radiofrequency (RF) ablation after completing the PFA lesion set.

To minimize procedure time, we preferred PVI and PWI with PFA over RF due to its time advantage. For example, Worck *et al*.^9^ reported a median time of 152 min to complete PVI and PWI using contact-force sensing guided RF ablation, while Schiavone *et al*.^10^ reported a median skin-to-skin time of 70 min for PVI and PWI with PFA. In our case, including the insertion of the Impella CP, the total skin-to-skin time was 121 min, with a left atrial dwell time of 48 min. Pulmonary vein isolation and PWI were achieved in 22 min. Although the total procedure duration was longer than in the aforementioned PFA study, there was still a time advantage over RF ablation.^9,10^

Other case reports on AF ablation in patients with tachycardia-induced cardiomyopathy requiring mechanical haemodynamic support have used either RF ablation or cryoballoon ablation for PVI.^3,11^ To our knowledge, this is the first case report of a patient on Impella support treated with PFA. Previously, it was unknown whether the Impella’s electromagnetic field could adversely affect the visualization and tracking of the pentaspline catheter. Conversely, it has not been evaluated whether the application of ultra-rapid electrical pulses interferes with Impella function. Since the Impella device’s motor unit has been reported to cause electromagnetic interference with the RHYTHMIA dual-sensor electroanatomic 3D mapping system, we chose the CARTO 3 electroanatomic mapping system for impedance-based catheter visualization, which proved feasible without any disturbances.^12^ Similarly, energy delivery at the pulmonary veins and posterior wall did not interfere with Impella function at any time.

## Conclusion

We present an example of the successful concomitant use of PFA for PVI and PWI alongside Impella CP in a haemodynamically unstable patient with acute heart failure caused by AF and AFLUT, without emergence of any inter-system interference. Haemodynamic stabilization through MCS enabled successful SVT ablation, thereby avoiding the need for AV-node ablation and permanent biventricular pacing.

## Data Availability

The data that support the findings of this study are available from the corresponding author upon reasonable request.

## Lead author biography

Dr Isabel Rudolph was born in Essen, Germany and graduated from University Duisburg-Essen in 2021. She worked as a resident in cardiology at the University Clinic Essen until March 2024. Since April 2024, Dr Rudolph continues residency at the Klinikum rechts der Isar in Munich. Her research has a focus on cardiac electrophysiology. Especially therapeutic strategies in the therapy of cardiac arrhythmias are a central topic of her research.

**Consent:** The authors confirm that written consent for submission and publication of this case report including image(s) and associated text has been obtained from the patient in line with COPE guidance.

**Funding:** None declared.
